# Study on the Influence Law of Dispersant on the Resistance Value of MWCNTs/PANI Epoxy Semiconducting Coatings

**DOI:** 10.3390/polym17233150

**Published:** 2025-11-27

**Authors:** Baiwen Du, Dengyun Li, Kai Zhu, Changxi Yue, Jia Xie, Ran Gao

**Affiliations:** 1 China Electric Power Research Institute Wuhan Branch, Wuhan 430000, China; dubaiwen@epri.sgcc.com.cn (B.D.); zhukai@epri.sgcc.com.cn (K.Z.); yuechangxi@epri.sgcc.com.cn (C.Y.); 2 State Grid Anhui Electric Power Co., Ltd., Hefei 230000, China; xiej5232@ah.sgcc.com.cn (J.X.); gaor4536@ah.sgcc.com.cn (R.G.)

**Keywords:** high-voltage DC voltage divider, semiconductor coating, resistance nonlinearity effect, PANI, MWCNTs, high molecular weight dispersant, equivalent resistance

## Abstract

Under the action of strong DC electric field, the resistance of the insulating cylinder of high-voltage DC voltage divider shows a nonlinear decrease with the increase in electric field strength, which leads to the decrease in measurement accuracy. It is shown that the effect can be suppressed to a certain extent by applying a semi-conductive coating on the surface of the insulating outer barrel. However, due to the inherent properties of the conductive nano-fillers, it is difficult to achieve uniform dispersion in the polymer matrix, which limits the electric field modulation ability of the coating and makes it difficult to fundamentally solve the nonlinear effect of the insulating outer barrel resistance. In this study, based on the multi-walled carbon nanotubes/polyaniline (MWCNTs/PANI) epoxy semiconducting coating system, the effects of dispersant type and dosage on the variation in the coating equivalent resistance with voltage were investigated. The semiconducting coatings with different dispersion states of conductive fillers were prepared by regulating the types and amounts of alkyl ammonium salt dispersants, polymer urethane dispersants, and low molecular weight unsaturated polycarboxylic acid polymer dispersants, and analyzed in combination with the equivalent resistance test, SEM microscopic morphology characterization, and coating adhesion test. The results show that the dispersion of the conductive filler has a significant effect on the suppression of the resistance nonlinear effect, and the polymer polyurethane dispersant can make the filler uniformly dispersed by virtue of the strong spatial site resistance, and the equivalent resistance change rate is lower than 28% under the additive amount of 0.5–1.5%, which has an excellent suppression of the nonlinear effect of the insulation resistance, and the adhesion of the coating is significantly improved to grade 0. The study provides theoretical basis and experimental support for the optimization of the preparation process of high-performance MWCNTs/PANI semiconducting coatings, which can help to improve the measurement accuracy and operational stability of high-voltage DC voltage dividers.

## 1. Introduction

High-voltage DC voltage divider is the key equipment for measuring DC high voltage [1,2]. Due to the combined effect of the strong DC electric field, it leads to the intensification of the phenomena of surface electron emission and air ionization along the surface, and the electronic conductivity and internal electrophoretic dissociation effects come to the forefront, which in turn triggers the phenomenon of nonlinear decrease in the resistance of the insulating outer cylinder. This results in a huge potential difference between the insulating cylinder and the internal resistance, leading to a dramatic increase in the measured current leakage and a significant decrease in the accuracy of the voltage divider [3,4]. Therefore, there is an urgent need to investigate the nonlinear effect of insulation resistance suppression methods to improve the voltage measurement accuracy.

Researchers have successfully suppressed the resistance nonlinearity effect of the insulation outer barrel of a high-voltage DC voltage divider by optimizing the structure of the voltage equalizing ring to regulate the electric field distribution. The China Electric Power Research Institute (CEPRI) successfully reduced the maximum field strength of the voltage divider from 51.49 kV/mm to less than 2 kV/mm by adjusting the size and position of the equalizing ring [5]; Wuhan University optimized the parameters of the equalizing ring by combining finite-element modeling, neural network, and the Gray Wolf algorithm, which resulted in a reduction in the maximum field strength of the 1100 kV device by 17.69% [6]. However, this method relying on insulation structure optimization has the obvious limitations of large space occupation and high cost. In contrast, the use of semiconducting coatings to modulate the electric field can improve the problem of uneven electric field distribution more flexibly and effectively and has economic and practical advantages [7]. Liang Hucheng’s team prepared epoxy resin/silicon carbide composites with nonlinear conductivity, explored the effect of nonlinear conductivity on the surface charge dissipation process, and reduced the nonlinear conductivity threshold to 2 kV/mm [8]. Sima Wenxia’s team prepared a nonlinear conductive film by doping a high concentration of ZnO and found that the nonlinear conductive film can reduce the electric field distortion and effectively inhibit partial discharges [9]. The ZnO/polyimide coating developed by ABB reduces the field strength at the edge of IGBTs to 6 × 106 V/m [10]. Semi-conductive coatings are an important means of regulating the electric field of high-voltage devices. Among them, multi-walled carbon nanotubes (MWCNTs) have a high aspect ratio, high electrical conductivity, and excellent mechanical properties, which can form an effective conductive network in the polymer matrix. On the other hand, polyaniline (PANI) not only has good conductivity, but also has environmental stability and dopability. Based on this, the group developed multi-walled carbon nanotubes/polyaniline (MWCNTs/PANI) epoxy semiconducting coating system, which significantly suppressed the insulation resistance nonlinear phenomenon. However, the high concentration of fillers is difficult to be uniformly dispersed in the composites, resulting in inhomogeneous material microstructure and increased interfacial defects, which can easily cause local conductivity anomalies in the materials, leading to weakening of the resistance nonlinearity suppression effect.

In this study, focusing on the insulation resistance nonlinearity of the casing, the role of the dispersion state of the conductive filler on the suppression of the resistance nonlinearity effect was investigated based on the multi-walled carbon nanotubes/polyaniline (MWCNTs/PANI) epoxy semiconducting coating system [11,12], which was successfully developed in the early stage of the research group, and which has the potential of electric field modulation. The semi-conductive coatings with different dispersion states of conductive fillers were obtained by modulating the type and amount of dispersant. Based on the equivalent resistance test platform, the suppression effect of the distribution characteristics of the conductive fillers in the semiconducting coatings on the nonlinear variation in resistance and the interfacial adhesion strength of the epoxy sleeve were evaluated. This study aims to provide theoretical basis and experimental support for the optimization of the preparation process of high-performance MWCNTs/PANI semiconducting coatings, which will ultimately serve to improve the measurement accuracy and operational stability of key equipment such as high-voltage DC voltage dividers.

## 2. Materials and Methods

### 2.1. Materials

The epoxy resin used in the study is bisphenol A diglycidyl ether provided by Dow Chemical (China) Investment Co., Shanghai, China, with an epoxy equivalent of 182~192 g/eq, and the curing agent, triethylenetetramine, is provided by Tokyo Kasei Kogyo Co., Tokyo, Japan. The conductive particles used polyaniline and multi-walled carbon nanotubes, were supplied by Shenzhen Suiheng Technology Co., Ltd. (polyaniline particle size < 30 μm, conductivity: 7.5 S/cm; multi-walled carbon nanotubes 8–25 nm in diameter and 5–15 μm in length), Shenzhen, China. The wetting dispersant, polymer chain dispersant, and controlled flocculation dispersant were provided by Bickel Chemistry, Burtelsdorf, Germany.

### 2.2. Methods

#### 2.2.1. Sample Preparation

**Figure 1:**
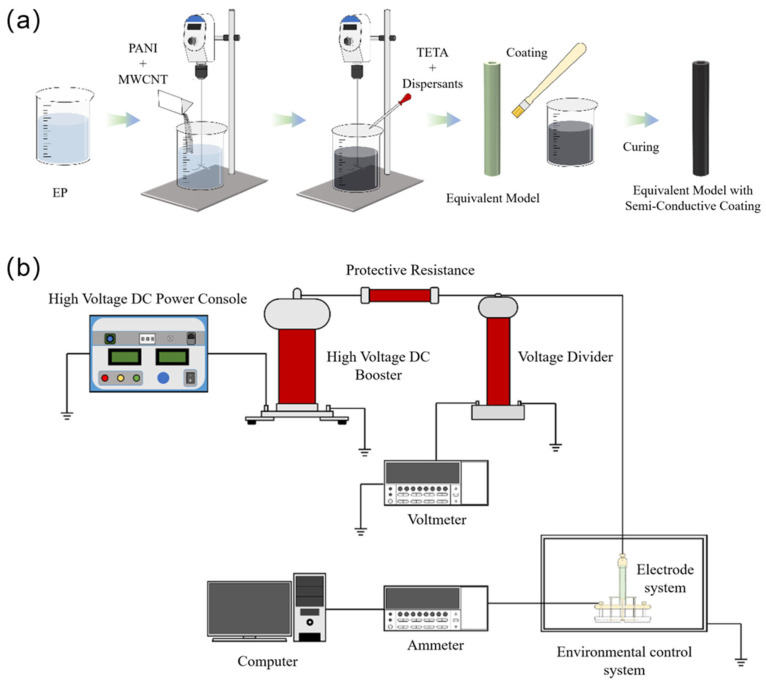
(**a**) Semi-conductive coating preparation process. (**b**) Equivalent resistance test platform.

The semi-conductive epoxy coatings and equivalent resistance samples were prepared according to the steps in Figure 1a. Firstly, bisphenol A diglycidyl ether was weighed into a beaker with a stirring rate of 200 r/min. Then, 2.5 wt% PANI and 0.5 wt% MWCNT of conductive filler were added, and the stirring rate was increased to 400 r/min for 2 h. Finally, triethylenetetramine and dispersing agent were added, and the amount of the triethylenetetramine was 13.04 wt%. Continuous stirring until the system components are fully mixed to obtain the semi-conductive epoxy coating. The semi-conductive coating was uniformly coated on the equivalent micro miniature sample, and cured at room temperature to obtain the sample coated with semi-conductive coating. The samples were named according to the dispersant type and dosage in accordance with EP, EP-0.5%A, EP-1%BYK-A, EP-1.5%A, EP-0.5%BYK-B, EP-0.5%BYK-B, EP-0.5%BYK-B, EP-0.5%BYK-C, EP-0.5%BYK-C, EP-0.5%BYK-C, respectively, wherein EP is an epoxy resin coating without adding dispersant, and A, B, and C are a wetting dispersant, a polymer chain dispersant, and a controlled flocculating dispersant, with additions of 0.5 wt%, 1 wt%, and 1.5 wt%, respectively.

#### 2.2.2. Equivalent Resistance Test

As shown in Figure 1b, the equivalent resistance test platform was used for the resistance test of the materials. The high-voltage DC power supply was NRZGF-200 kV/5 mA series high-voltage power supply, and the protective resistors used were vitreous enamel film high-voltage resistors. The DC high-voltage divider used has a dividing ratio of 1000 and a rated voltage of 200 kV with an accuracy of 1%. The voltmeter is a Keithley 6517B electrostatic meter (Tektronix, Cleveland, OH, USA). The voltmeter is connected to the low voltage arm of the DC high-voltage divider so that its indication is in the ratio of 1:1000 with the real voltage applied to the sample, by which the voltage applied to the sample is measured and the output voltage of the high-voltage DC voltage is adjusted according to the indication of the voltmeter. The ammeter used to measure the current through the sample is a Keithley 6485 Picoammeter (Tektronix, Cleveland, OH, USA), with a measurement accuracy of 10–14 A and a range of ±10 fA–±21 mA. The ammeter is connected to the computer and can communicate with the computer, which automatically collects and saves the test data. The environmental control system is used to simulate the working environment of the UHV DC voltage divider. In this paper, the temperature condition is set to 25 °C and the humidity condition is set to 40% RH when testing the insulation resistance of the equivalent miniature sample.

#### 2.2.3. SEM

In this study, a Zeiss Sigma 500 field emission scanning electron microscope (ZEISS, Oberkochen, Germany)was used to characterize the microscopic morphology of the composites. The specimen preparation followed the GB/T 1040.2-2022 standard [13], and the standard dumbbell-type specimen was prepared by injection molding process. In order to obtain the ideal observation cross-section, the specimen was brittle fracture processed by liquid nitrogen deep cooling, and the cross-section morphology was observed after gold spraying by ion sputtering instrument.

#### 2.2.4. Coating Adhesion Test

In accordance with the national standard GB/T 9286-1998 [14] “color paints and varnishes, scratching test”, according to the sample substrate and the thickness of the paint film with different spacing of the scratching tool on the paint film for the grid pattern cutting, so that it is exactly penetrated to the substrate, according to the area of the paint film from the scratching area of the substrate off to determine the level of adhesion.

## 3. Results and Discussion

**Figure 2:**
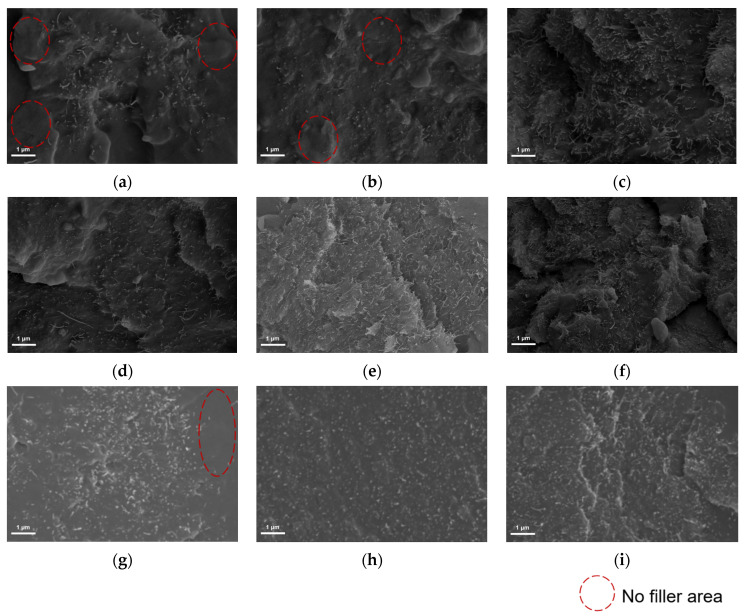
Cross-sectional morphology of epoxy materials: (**a**) EP-0.5%A; (**b**) EP-1%A; (**c**) EP-1.5%A; (**d**) EP-0.5%B; (**e**) EP-1%B; (**f**) EP-1.5%B; (**g**) EP-0.5%C; (**h**) EP-1%C; (**i**) EP-1.5%C.

The effects of wetting dispersant, polymer chain dispersant, and controlled flocculation dispersant on the dispersibility of conductive filler were investigated separately. The main component of the wetting-type dispersant is alkyl ammonium salt, which can interact with the filler surface through the hydrophilic groups in its molecule to improve the surface wettability of the filler, reduce the interfacial energy between the filler and the substrate, and promote a good contact between the filler and the substrate so as to reduce the agglomeration of the filler [15]. Figure 2a–c give the scanning electron microscopy microcharacterization results of the semiconducting coating after the addition of the wetting dispersant. It can be seen that for sample EP-0.5%A and sample EP-1%A, due to the insufficient amount of dispersant addition, the distribution of conductive fillers in the coatings is not uniform, and a large number of non-conductive filler zones exist. With the increase in the additive amount, the wetting dispersant improves the dispersion state of the filler, and when the additive amount reaches 1.5%, the uniform distribution of the filler is realized and the agglomeration phenomenon is reduced. Through Figure 2d–f, it can be seen that the conductive filler in the sample can realize uniform distribution after the addition of polymer chain dispersant. The main component of the polymer chain dispersant is polyurethane solution, which contains long-chain polymers with a strong spatial site-blocking effect, can effectively prevent the filler particles from contacting each other. Spatial site resistance ensures the uniform dispersion of filler by forming a physical barrier on the filler surface so that the filler particles cannot aggregate with each other, thus ensuring the uniform dispersion of the filler [16]. The main component of controlled flocculating wetting and dispersing agents is a low molecular weight unsaturated polycarboxylic acid polymer solution, which has the ability to both wet the filler and regulate the flocculating effect according to the change in the amount added. Figure 2g–i give the dispersion effect of the conductive filler in the coating after the addition of the controlled flocculating dispersant, and it can be seen that the conductive filler in EP-0.5%C failed to achieve a uniform distribution, and there was a no filler distribution region in the matrix, and the conductive filler in EP-1%C, EP-1.5%C was well dispersed.

**Figure 3:**
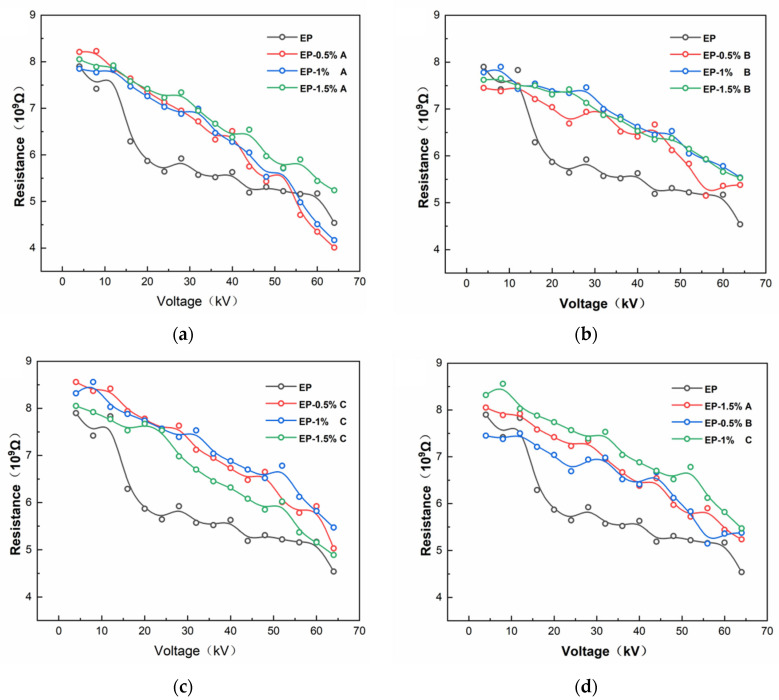
Variation in equivalent resistance with voltage. (**a**) Effect of wetting dispersant content on equivalent resistance. (**b**) Effect of polymer type dispersant content on equivalent resistance. (**c**) Effect of controlled flocculation type dispersant content on equivalent resistance. (**d**) Effect of dispersant type on equivalent resistance.

As shown in Figure 3, the equivalent resistance of the micro models with different dispersant types and dosages were measured using the equivalent resistance test platform, respectively, and the test voltage interval was 4–64 kV. It can be seen that the equivalent resistance of the samples showed a decreasing trend with the increase in the applied voltage level. Among them, the variation in equivalent resistance with voltage for EP samples is 42%. The dispersant can significantly affect the stability of the equivalent resistance by regulating the dispersion state of MWCNT and PANI. After adding the wetting dispersant, as shown in Figure 3a, the variations in equivalent resistance with voltage were 51% (EP-0.5%A), 46% (EP-1%A), and 34% (EP-1.5%A), respectively. The change in equivalent resistance of the wetting dispersant was as high as 51% at 0.5% addition. This is caused by the uneven distribution of MWCNT agglomerates and PANI due to the insufficient addition of wetting dispersant, forming local gaps in the epoxy resin matrix. When the additive amount was raised to 1.5%, the filler surface was fully wetted, which significantly improved the dispersion of the filler. The MWCNT and PANI are dispersed more uniformly, which in turn reduces the equivalent resistance change rate to 34%, effectively improving the inhibition effect of the semiconducting coating on the nonlinear change in the equivalent resistance. After the addition of polymer dispersant, as shown in Figure 3b, the variation in equivalent resistance with voltage was 27% (EP-0.5%B), 28% (EP-1%B), and 27% (EP-1.5%B), respectively. This is due to its anchoring on the filler surface, it will form a thick and dense polymer layer on the filler surface. When the fillers are close to each other, the polymer layer is compressed and produces a strong repulsive force to avoid the fillers from approaching further. By virtue of the strong spatial resistance formed by the long-chain polymer, the filler is uniformly dispersed at all additive amounts, and the MWCNT is effectively untangled, so that the equivalent resistance change rate is always lower than 28%, which is optimal for the suppression of nonlinear phenomena. After the addition of controlled flocculating dispersant, as shown in Figure 3c, the variations in equivalent resistance with voltage were 41% (EP-0.5%C), 34% (EP-1%C), and 39% (EP-1.5%C), respectively. The dispersant was able to introduce charged groups on the surface of the filler, which produced repulsive forces when two identically charged particles approached, preventing the particles from approaching further. The controlled flocculating dispersant has the best charge balance at 1% addition, and the rate of change is reduced to 34%; however, insufficient charge repulsion at low addition leads to agglomeration, and excess ions at high addition triggers electric field distortion, which increases the rate of change in the equivalent electrical resistance to 41% and 39%, indicating that its stability is susceptible to voltage interference. In summary, the phenomenon of filler agglomeration significantly affects the rate of change in equivalent resistance with voltage, and when agglomeration of the conductive filler in the coating occurs, the filler is not uniformly distributed in the coating, leading to the formation of high conductivity channels in localized regions [17]. These high conductivity regions are more likely to become the main channels for carrier transport under the action of electric field, which makes the change in resistance have a large nonlinearity, which in turn leads to a reduction in the ability of the coating to suppress the nonlinear effect of the equivalent resistance. Therefore, it is important to ensure good dispersion and uniform distribution of fillers to suppress the resistance nonlinear effect.

**Figure 4:**
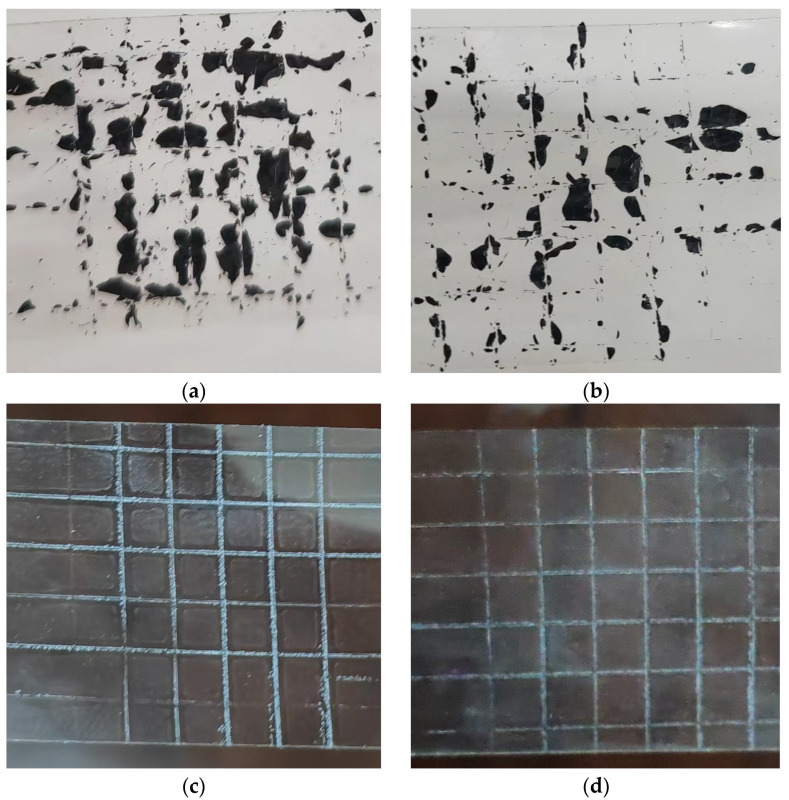
Adhesion of semi-conductive coatings containing different dispersants. (**a**) No dispersant epoxy coating; (**b**) 1.5 wt% wetting dispersant epoxy coating; (**c**) 0.5 wt% polymer dispersant epoxy coating; (**d**) 1 wt% wetting controlled flocculating dispersant epoxy coating.

The effect of dispersant on the distribution of conductive filler in the composites will further have an impact on the adhesion of the semiconducting coatings; therefore, the coating adhesion test was conducted. As shown in Figure 4a, the semiconducting coating samples without added dispersant showed severe peeling phenomenon in the peeling test, and the adhesion grading was about 4, indicating that the bonding between the coating and the substrate was weak and the coating was easy to peel off. When a wetting dispersant was added, Figure 4b, although the wettability and dispersibility of the conductive filler could be improved, the adhesion of the coating failed to be significantly improved, and the peeling phenomenon was more serious in the peeling test, and the adhesion grading was still at level 4. This is because the wetting dispersant improves the dispersibility of the filler, but its main role is to improve the distribution of the filler in the substrate, and it fails to effectively enhance the interaction force between the coating and the substrate. It shows that the wetting dispersant mainly relies on the change in surface tension to improve the dispersion of filler, but for the enhancement of adhesion between the filler and the substrate, its effect is relatively weak. When the polymer dispersant and controlled flocculating dispersant were added, the adhesion of the coatings in Figure 4c,d was significantly improved, and the coatings did not show obvious peeling phenomenon in the peeling test, and the grading reached grade 0, which showed excellent adhesion. The reason is that these two dispersants not only can effectively improve the dispersion of conductive fillers and avoid the phenomenon of filler agglomeration, but also enhance the bonding force between the coating and the substrate through different physical and chemical action mechanisms. Polymer dispersant through the long-chain polymer forms a strong spatial resistance effect, preventing the filler agglomeration, so that the filler can be uniformly dispersed in the coating; at the same time, the chemical structure of the polymer chain and the physical properties of the coating and the substrate enhance the adhesion between the coating and the adhesive force. This strong spatial site-blocking effect is also able to provide some mechanical locking cooperation, which makes the coating adhere more firmly to the surface of the substrate [18]. Similarly, controlled flocculating dispersants exhibit a similar effect. By introducing negatively charged dispersant molecules into the coating, not only is the dispersion of the filler improved, but also the adhesion between the coating and the substrate is strengthened through electrostatic interactions. Since the controlled flocculating dispersant is able to form a more stable charge balance between the coating and the substrate, it provides a chemical basis for enhancing the mechanical adhesion of the coating while providing good dispersibility.

The stability of the semiconducting coating under DC ultra-high voltage is the key to the semiconducting coating for the standard device insulation structure resistance nonlinear governance. The standard device insulation structure should have a small resistance voltage coefficient after coating the semiconducting coating in order to prevent the insulation structure resistance nonlinear drop caused by the standard device measurement misalignment. Voltage Coefficient of Resistance (VCR) refers to the degree of change in resistance value with the change in voltage. It can be expressed by the following formula,

(1) $$VCR=\frac{1}{R}\;\frac{∆R}{∆V}$$

where *R* is the base value of the resistor; Δ*R* is the value of the change in resistance, Δ*V* is the amount of change in voltage across the resistor; and the unit of *VCR* is ppm/V.

**Figure 5:**
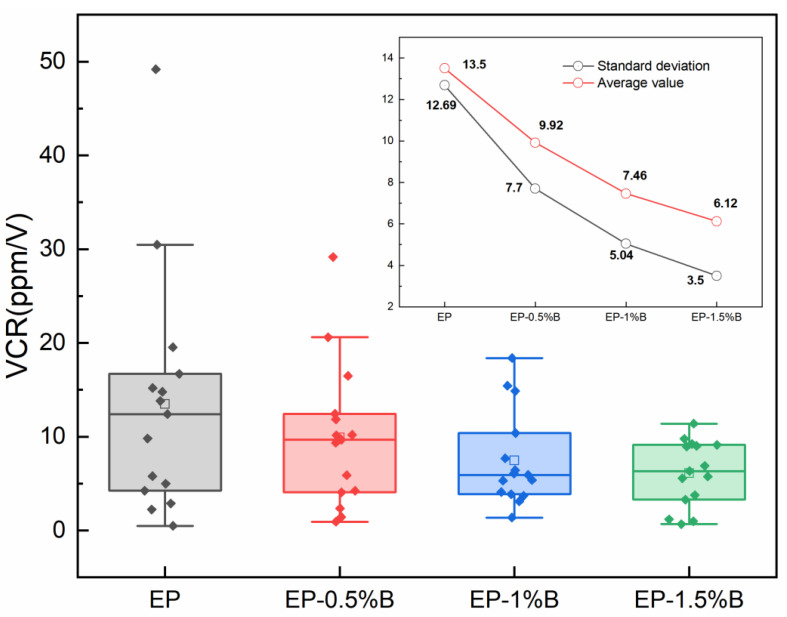
Effect of polymer-based dispersant content on voltage resistance coefficient.

Based on the results of the equivalent resistance test, the resistance–voltage coefficients of the dispersant-unadded and polymer-based dispersant formulation systems were calculated, and the results are shown in Figure 5. It can be seen that with the increase in dispersant content, the mean and standard deviation of the sample VCR are significantly reduced, showing an overall decreasing trend, and the EP-1.5%B sample has the lowest VCR value, indicating that its resistance is more stable. This is due to the fact that the addition of dispersant significantly improves the dispersion uniformity of the conductive filler in the semiconducting coating compared to the sample without dispersant. In the composites with uniform distribution of conductive fillers, the MWCNTs particles were equilibrated in terms of spacing and were isolated by PANI particles. With the increase in applied voltage, although electrons gained energy to cross the potential barriers for jumping, the weakly conducting PANI formed a barrier to the electron migration, weakening the contribution of field emission effect to the resistance decrease, which led to the decrease in the absolute value of VCR. On the contrary, in materials with inhomogeneous filler distribution, the agglomeration phenomenon leads to incomplete conductive pathways. At high voltages, the localized strong electric field formed between the agglomerates makes it easier for electrons to cross the interfacial barriers and jump, while the high-density distribution within the agglomerates exacerbates the overdiffusion and tunneling effects, resulting in a sharp decrease in resistance with voltage, which is manifested in a larger VCR value.

## 4. Conclusions

Based on the equivalent resistance test platform, this paper explores the dispersion behavior of conductive fillers in semi-conductive coatings on the nonlinear effect of coating resistance suppression by performing equivalent resistance tests on micro models with different dispersant types and dosages, and draws the following important conclusions:

(1) The dispersion of conductive fillers in semiconducting coatings has a significant influence on the effectiveness of suppressing the resistive nonlinear effect. The degree of dispersion of the filler directly determines the rate of change in electrical resistance of the coating at different voltages. When the conductive filler is uniformly distributed in the coating, the coating is more effective in suppressing the resistive nonlinear effect, and the resistive rate of change is lower. When the filler agglomeration phenomenon, the abnormal increase in local conductivity leads to the enhancement of the resistance nonlinear effect of the coating.
(2) The addition of dispersants can effectively solve the uneven distribution of conductive fillers in the coating. Among them, polymer disperses through the spatial site resistance effect of long-chain polymers, effectively avoiding the phenomenon of filler agglomeration, promoting the uniform distribution of fillers in the coating; compared with wetting dispersants and controlled flocculation dispersants, its effect is better, using only 0.5% of the additive amount to achieve uniform dispersion of conductive fillers in the coating.
(3) The interaction between the polymer dispersant and the coating substrate through the chemical structure of its long-chain polymer enhances the adhesion between the coating and the substrate surface and significantly reduces the occurrence of coating peeling phenomenon.

## Data Availability

The original contributions presented in this study are included in the article. Further inquiries can be directed to the corresponding author.
